# Monitoring of Non-Maximum-Residue-Level Pesticides in Animal Feed: A Study from 2019 to 2023

**DOI:** 10.3390/toxics12090680

**Published:** 2024-09-19

**Authors:** Roberta Giugliano, Vittoria Armenio, Valentina Savio, Erica Vaccaro, Valentina Ciccotelli, Barbara Vivaldi

**Affiliations:** National Reference Laboratory of Pesticides in Cereals and Feed (NRL), Istituto Zooprofilattico Sperimentale del Piemonte, Liguria E Valle D’Aosta, Piazza Borgo Pila 39/24, 16129 Genoa, Italy; vittoria.armenio@izsto.it (V.A.); valentina.savio@izsto.it (V.S.); erica.vaccaro@izsto.it (E.V.); valentina.ciccotelli@izsto.it (V.C.); barbara.vivaldi@izsto.it (B.V.)

**Keywords:** pesticides, feed, maximum residue levels (MRLs)

## Abstract

Pesticides play a critical role in modern agriculture by protecting crops and ensuring higher yields, but their widespread use raises concerns about human health and environmental impact. Regulatory agencies impose Maximum Residue Levels (MRLs) to ensure safety, and the European Food Safety Authority (EFSA) assesses pesticide risks. This study monitored pesticide residues in 169 feed samples from Piedmont (Italy) collected between 2019 and 2023. Using GC-MS/MS, residues were found in 92% of animal-based and 70% of cereal-based feedstuffs. The most common pesticides in cereal-based feeds were pyrimiphos-methyl, deltamethrin, cypermethrin, azoxystrobin, and tetramethrin, and the pesticide synergist piperonyl-butoxide demonstrated a significant increase in contaminated samples in 2023. The lower concentrations in 2021 were likely due to COVID-19 impacts on pesticide availability. In animal-based feeds, common pesticides included deltamethrin, cypermethrin, and the pesticide synergist piperonyl-butoxide. The results highlight the pervasive presence of low-dose pesticide mixtures in feed and food chains, which could impact health, although do not pose acute risks. The study emphasizes the need for ongoing pesticide monitoring and awareness of the long-term effects of chronic pesticide exposure on animal, human, and environmental health.

## 1. Introduction

Pesticides are chemical substances or biological agents used to prevent, control, or eliminate pests, including insects, weeds, fungi, and other organisms that can damage crops and reduce agricultural productivity. They play a crucial role in modern agriculture by protecting crops from various threats, ensuring higher yields and reducing losses caused by pests and diseases [1,2]. The use of pesticides helps farmers to maintain crop integrity and increase food production, which is essential for meeting the growing global food demand [2,3]. However, the widespread application of pesticides raises concerns about their potential impact on human health and the environment, as the molecular targets of pesticides are often shared between pest and non-target species, including humans [4].

Regulatory agencies impose Maximum Residue Levels (MRLs) for each pesticide in food and animal feedstuff commodities [5,6]. MRLs are legally permitted maximum concentrations in food and animal feed [7,8]. The European Food Safety Authority (EFSA) plays a crucial role in evaluating the safety of pesticides by providing scientific advice and conducting risk assessments [9]. These assessments provide scientific opinions, which are useful for establishing new MRLs [9].

Among synthetic pesticides, the major classes are organochlorines, organophosphates, carbamates, and pyrethroids. Organochlorine pesticides (also called chlorinated hydrocarbons) are organic compounds with five or more chlorine atoms; organophosphorus pesticides are phosphoric-acid-derived pesticides; carbamates are organic pesticides derived from carbamic acid; and pyrethroids were originally organic compounds isolated from the naturally occurring flowers of pyrethrums. And pyrethroids can be subdivided into two categories based on their toxic effects: Type I and Type II [10].

For feedstuff matrices, European laws fix MRLs (Maximum Residue Levels) for organochlorine pesticides (CE 32/2002 and CE 396/2005), but no restrictions are fixed for other pesticide compounds [5,6].

Studies on pesticides’ health effects have highlighted the neurotoxic activity of organochlorine, organophosphate, and pyrethroid pesticides [4]. Recent studies involving human cohorts have found positive correlations between the consumption of organic food and a reduced incidence of obesity, cancer, and various other diseases [11,12,13,14,15,16,17,18]. In a pioneering murine study, scientists fed rats with organic and conventional crops and observed the differences between the two groups in terms of growth, hormonal, and immune system parameters, which are known to affect the risk of several chronic, non-communicable diseases [18]. Today, the assessment of mixture effects is still often conducted using binary mixtures of similarly acting pesticides [18]. However, given the immense number of potential mixture combinations, the resulting effects of pesticide mixtures can vary widely [18,19]. Detrimental effects can occur even with chronic low doses of pesticides, as demonstrated by Sheer, Charli, and colleagues [20,21]. They noted that chronic nano- or micro-molar concentrations of pyridaben in cultured neuronal cells and organotypic midbrain slices induced significant neurotoxic effects [20,21].

Furthermore, the excessive use of pesticides also results in environmental challenges [3,4,10,11,12,13,14,15,16,17,18,19,20,21,22,23,24,25,26]. Lately, the European Green Deal, in conjunction with the Farm to Fork and Zero Pollution strategies, has set ambitious targets: reduce pesticide usage by 50%, eliminate soil pollution, and ensure at least 25% of the farmland in Europe is organic by 2030 [25,27,28]. Achieving these objectives requires a thorough assessment of the current situation and the regular monitoring of pesticide use across various segments of the feed and food chains. Significant uncertainties remain regarding the true pesticide contamination of the environment, the dynamics of this contamination, and the contamination risks to the food chain [29,30]. Pesticides can be found in animal-derived foods such as milk, eggs, honey, and meat, as well as in organs after slaughter [24,31]. According to the EFSA, 12.8% of 14,439 animal samples contained quantifiable concentrations of contaminants at or below the Maximum Residue Limit (MRL), likely due to the ingestion of contaminated feed [24,31,32]. In “The 2021 EU Report on Pesticide Residues”, the EFSA highlights the presence of chlordecone, an obsolete organochlorine pesticide also known as Kepone, in chicken eggs. This contamination is attributed to the persistence of chlordecone in soil, affecting chickens in open-cage farms where feed is exposed to previously treated soil [32]. Moreover, pesticide carryover poses a potential risk to all organic crops, making it currently impossible to achieve a ‘zero-tolerance’ policy [3]. Once ingested by livestock through feed, these pesticides are excreted via faeces, impacting surrounding ecosystems [26].

A study published in Scientific Reports observed the negative effects of routine cattle health treatments containing triclabendazole and synthetic pyrethroids on the abundance of dipteran larvae in bovine faeces [26]. This study aimed to understand the decline in red-billed chough (*Pyrrhocorax pyrrhocorax*) populations and found that treatments using triclabendazole and deltamethrin significantly reduce arthropod larvae in faeces, underscoring the broader ecological consequences of pesticide use [26].

In our opinion, despite the holistic and multidimensional approach of One Health, most attention has been focused on the interactions between animal and human health, with considerably less emphasis on environmental and plant health. However, there is increasing evidence that the challenges of climate change, food and nutritional insecurity, and biodiversity loss can be most effectively addressed within the One Health framework, as stated in the “Berlin Principle of One Health” [22,23].

Based on recent findings regarding the neurotoxic effects of pesticides [4,10,11,12,19,20,21] and the interconnectedness and interdependence of human, animal, plant, and environmental health emphasized by the One Health approach [22,23], we propose monitoring not only banned pesticides in feedstuff, but a wider panel of pesticides. The present monitoring study takes advantage of the same equipment and procedures employed in routine analysis, but investigates a broader GC-MS/MS panel list than the banned list of CE 32/2002 and CE 396/2005. This suggested monitoring will provide a wider overview of pesticide contamination and, if necessary, authorities could implement plans to quickly detect the concentrations of non-MRL pesticides before they can enter the food chain. Indeed, as reported by others, such early detection is crucial to preventing neurotoxic diseases in animals, and then in humans once animals are slaughtered [3,4,10,11,12,13,14,15,16,17,18,19,20,21,22,23,24,25,26,31].

## 2. Materials and Methods

### 2.1. Sample Collection

To evaluate chemical safety, research was conducted on a representative selection of cereal-based and animal-origin-based feedstuff sampled from a representative number of Northwestern farms. Sampling was conducted following the Annual National Residue Plan (PNR 2023), which is based on risk analysis. The sampling plan was chosen based on Health Ministry and EFSA guidelines to ensure complete control of the samples’ trading and use in routine farm activity. The plan is implemented through three strategic plans: (1) the Targeted Plan, which is based on risk and verifying the compliance of feeds produced in member states; (2) The Surveillance Plan, which involves random monitoring for a wide range of substances; and (3) the Third Country Plan, in case of Third Country importation. Activities outside of these plans (i.e., “Extrapiano”) can be planned by the ministry or regional/local authorities for specific national or local needs, such as additional control activities, under specific conditions. These four activities are planned and reported in the NSIS/RaDISAN system. Suspicion-based activities, which are not planned, must also be reported in the NSIS/RaDISAN system as per the guidelines [33].

In total, 169 feed samples were obtained during 2019–2023. Of the investigated samples, 108 were cereal-based and 61 were animal-origin-based. All fresh matrices were kept in a fridge at a controlled temperature (2 ÷ 8 °C) for no more than 5 days; meanwhile, dried matrixes were kept at room temperature for no more than 2 weeks. For all dried matrices, the humidity percentage was registered, and the final concentration was obtained by correcting for the humidity percentage registered before the sample analysis, following this equation (Equation (1)):

(1) $$C_{fin}=\frac{C*88\%}{\left(100-H\right)}\;$$

where *C_fin_* is the analyte concentration (ppm) at 12% humidity; *C* is the concentration obtained by the GC analysis; *H* is the humidity (%) registered before sample analysis, and88 is obtained considering the 100% of humidity minus 12%.

### 2.2. Chemicals

All pesticide standards, reagents, and solvents were purchased from Merck (Darmstadt, Germany). Below are listed the pesticides monitored: 2-Pheylphenol, Azoxystrobin, Beta-Endosulfan, Biphenyl, Bixafen, Boscalid, Chlorpyrifos, Chlorpyrifos-methyl, Cypermethrin, Cyprodinil, Deltamethrin, Desmedipham, Difenoconazole, Diphenylamine, Etofenprox, Fenazaquin, Fenthion, Fenvalerate, Fipronil, Fludioxonil, Lambda-Cyhalothrin, Malaoxon, Malathion, o, p′-DDT, p, p′-DDE, Pendimethalin, Permethrin, Phosalone, Piperonyl-butoxide, Pirimiphos-methyl, p, p′-DDD + o, p′-DDT, Procymidone, Pyridaben, Pyrimethanil, Tau-Fluvalinate, Tetramethrin, Triadimenol, and Trifluralin. Triphenylphosphine (TPP) was used as the internal standard.

### 2.3. Sample Preparation and Instrument Parameters

All samples were grounded and homogenized. Cereal-based samples were treated using the SweEt method [34,35]; meanwhile, for animal-origin samples, the QuEChERS method was used. The SweEt method consists of adding water and extracting with ethyl acetate (added with 1% acetic acid). Then, separate the two phases by centrifugation and inject the supernatant without any purification step. The QuEChERS method consists of adding acetonitrile, extracting using QuEChERS salts (4 g of magnesium sulphate, 1 g of sodium chloride, 1 g of trisodium citrate dihydrate, and 0.5 g of disodium hydrogen citrate sesquihydrate), and purifying using d-SPE salts (900 mg of magnesium sulphate, 150 mg of PSA, and 150 mg of C18). All extracts were injected into a GC-MS/MS system (Thermo SCIENTIFIC TRACE 1300 coupled with TSQ 8000 Evo, Waltham, MA, USA) equipped with an AS 3000 autosampler, following the detailed methodology reported in our previous work [35]. Blank reagents, blank matrices (internally produced), and fortified matrices were analysed in every analytical batch. Concentrations for the matrix curves were as follows: 0.5, 1, 2, 5, and 10 ppm.

Mass transition information is reported in Tables S1 and S2 in the Supplementary Material. The matrix effect results have been validated and are reported in Table S3.

## 3. Results and Discussion

The analyses revealed that 56 animal-based and 76 cereal-based feedstuffs contained pesticide residues over the LOQ. Considering a confidence level of 95%, the sample size chosen allows a margin of error of approximately 6.53%, which, in our opinion, can provide a broad overview of feed safety in this pilot study.

**Figure 1:**
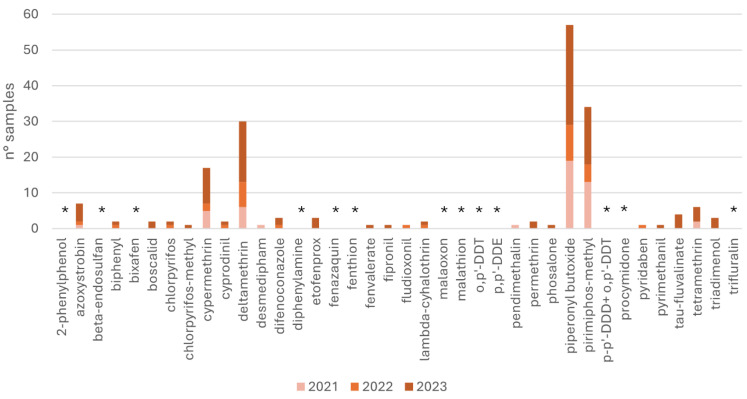
Number of cereal-based feedstuff samples contaminated by pesticides in 2021, 2022, and 2023. Asterisk refers to compounds not detected in any samples throughout the three years.

In cereal-based feed, azoxystrobin (n = 7), cypermethrin (n = 17), deltamethrin (n = 30), piperonyl-butoxide (n = 57), pyrimiphos-methyl (n = 34), and tetramethrin (n = 6) were the most common pesticides and synergists. Figure 1 shows the number of samples contaminated by pesticides detected from 2021 to 2023.

**Figure 2:**
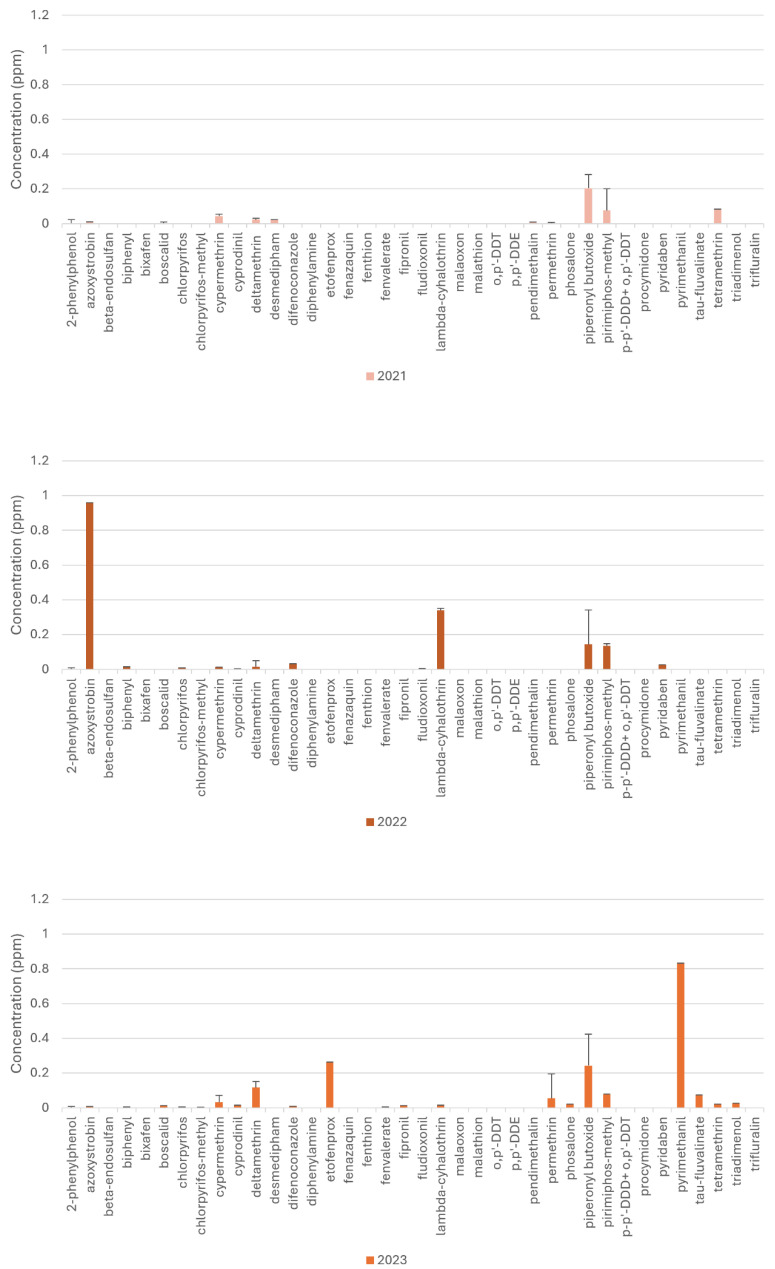
Annual trends of concentrations of residues detected from 2021 to 2023 in cereal-based feed.

The highest residues were detected in straw (azoxystrobin 0.96 mg/kg), in fodder (cypermethrin 0.143 mg/kg), in oat-based feed (deltamethrin 1.396 mg/kg), in ground corn (piperonyl-butoxide 1.505 mg/kg), in durum wheat bran (pyrimiphos-methyl 0.562 mg/kg), and in a complete feed (tetramethrin 0.083 mg/kg). Figure 2 shows the annual trends of the concentrations of residues detected from 2021 to 2023 in cereal-based feed.

**Figure 3:**
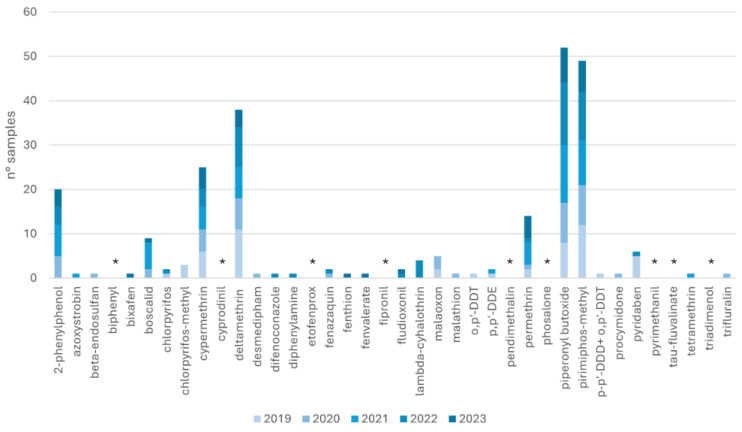
Number of animal-origin-based feedstuff samples contaminated by pesticides from 2019 to 2023. Asterisk refers to compounds not detected in any samples throughout the three years.

On the other hand, in animal-based feed, 2-phenylphenol (n = 20), boscalid (n = 9), cypermethrin (n = 25), deltamethrin (n = 38), permethrin (n = 14), piperonyl-butoxide (n = 52), piperonyl-methyl (n = 49), and pyridaben (n = 6) were the most common pesticides and synergists. During 2019, the highest piperonyl-butoxide residue was found in complete feed for trout (1.08 mg/kg). Figure 3 shows the number of samples contaminated by pesticides detected from 2019 to 2023.

As illustrated in Figure 1, the statistical analysis revealed that the number of cereal-based samples with detectable pesticides was higher in 2023 compared to 2022 and 2021, as confirmed by the Kruskal–Wallis test (*p* < 0.05). Meanwhile, no statistical difference was reported among the number of animal-based samples investigated from 2019 to 2023 (*p* > 0.05) (Figure 3). Additionally, most of the pesticides monitored in 2021 had the lowest averages (79% of the entire panel monitored in animal-origin feeds and 53% monitored in cereal-based ones). These findings are consistent with the Annual European Union report on pesticide residue in food [32,36]. In 2020, the EFSA registered 94.9% of the overall 88,141 samples analysed as below the MRL and 5.1% as over the MRL, of which 3.6% were non-compliant; meanwhile in 2021, 96.1% of the overall 87,863 samples analysed were below the MRL and 3.9% were over the MRL, of which 2.5% were non-compliant [32,36]. To the best of our knowledge, in the literature there are no similar studies that compare pesticide residue by years around the COVID-19 period. Further studies are necessary to validate our hypothesis [37,38]. However, the minimal residues observed in 2021 might not be attributable to COVID-19 but to other factors not considered in the study. These factors could include the varying sources of raw materials used in the feed or the presence of other pesticides not included in the study panel, or other confounding factors not considered here.

Comparing the residue concentrations in the cereal-based samples shown in Figure 2 revealed a significant difference between all the periods investigated; in particular, lower concentrations were detected during 2021, as confirmed by the Kruskal–Wallis test (*p* < 0.05). The press reported that the lockdown and quarantine measures implemented to combat the COVID-19 pandemic restricted the production and supply of PPPs, significantly impacting crop protection activities worldwide [39,40,41,42,43]. In 2021, a laboratory analysed samples of feed produced with raw materials grown the previous year, during the limited availability of pesticide and synergistic solutions, as reported by other authors and journals [39,40,41,42,43]. So, authors suggest that the lower concentration detected during 2021 could be a result of the challenges posed by the COVID-19 pandemic.

**Figure 4:**
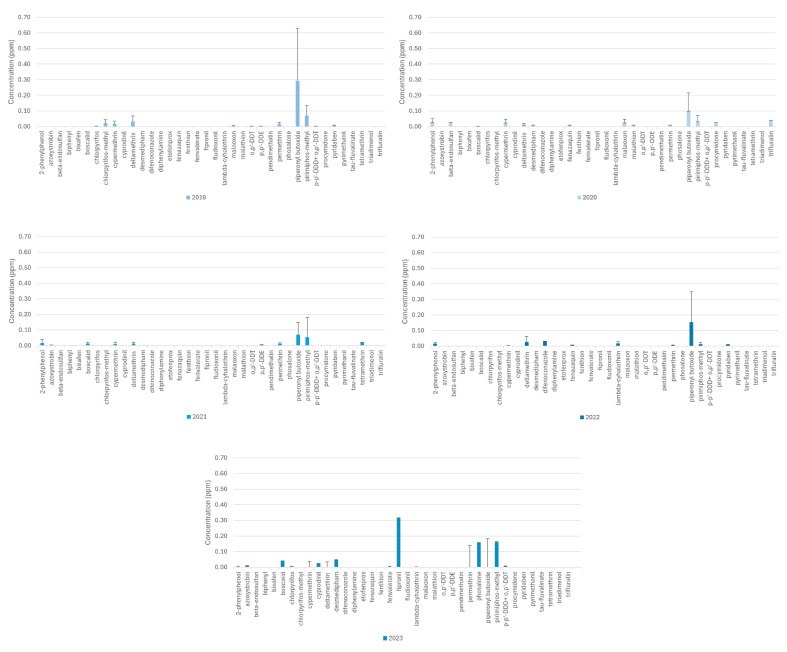
Annual trend of the concentration of the residues detected from 2019 to 2023 in animal origin-based feed.

As shown in Figure 2 and Figure 4, most of the residues detected belong to the family of insecticides pyrethroids. Raw data are reported in Table S4 in the Supplementary Material. Among the detected compound residues, six residues (permethrin, phosalone, piperonyl butoxide, pirimiphos-methyl, tetramethrin, and triadimenol) are not approved as plant protection products for the European Union’s market by the Regulation (EC) 1107/2009 [24,44,45]. These findings are consistent with those reported in an Austrian study, as confirmed by Student’s *t*-test (*p* < 0.05). The first Austrian study examining a broad spectrum of pesticides and veterinary drugs in the diets of dairy cattle detected metolachlor, piperonyl butoxide, pirimiphos-methyl, and diethyltoluamide residues [24]. More than 90% of the diets contained pesticide residues, although banned pesticides were not found [24]. In particular, residues were detected in by-products of beer production intended to be incorporated in dairy cow diets [24,45]. These findings highlight the prevalent presence of low-dose pesticide mixtures in the feed and food chains, especially when by-products from the food industry are incorporated [45]. As reported by Palladino et al., barley, which is a cereal mostly used for beer production, is frequently contaminated by pesticides [38]. Furthermore, evidence from the literature suggests that pesticide residues have an impact on the health of animals, humans, and the environment [24]. Various strategies have been implemented to monitor the presence of substances harmful to humans and the environment, and feed analysis can be very useful, especially in terms of time efficiency. Evidence of the long-range transport of agrochemicals through air currents and atmospheric transfer from agricultural fields is reported by many American studies [37,46,47,48]. This transfer can be harmful as it leads to the spread of pesticides. This potential carry-over poses a health risk because pesticides not specific to a certain crop may still be present on the plant, unbeknownst to both the producer and the consumer [37,46,47,48].

In animal origins-based feedstuff no significant difference among the residue concentrations was detected among all years investigated (*p* > 0.05) (Figure 4). The source of these pesticide contaminations was likely the ingestion of contaminated feed [24,31,32]. However, animals metabolize and degrade pesticides producing pesticide metabolites [31,49,50]. Therefore, the presence of pesticides in animal tissues does not fully reflect the residues level and mixture originally present in their feed [31,32].

## 4. Conclusions

The analysis showed that 92% of animal-based and 70% of cereal-based feedstuffs contained pesticide residues above the limit of quantification (LOQ). Banned pesticides in feedstuff are listed in CE 32/2002 and CE 396/2005, and consist of organochlorine pesticides, which are monitored using GC-MS/MS. In this study, we proposed a monitoring study using the same equipment and procedures employed in routine analysis, but for a broader GC-MS/MS panel list. 

In cereal-based feeds, the most common pesticides were pyrimiphos-methyl, deltamethrin, cypermethrin, azoxystrobin, tetramethrin, and a pesticide synergist, i.e., piperonyl-butoxide. A significant increase in the number of contaminated cereal-based samples was registered in 2023 compared to 2022 and 2021. The authors suggest that the lower residue concentrations in 2021 were likely due to the COVID-19 pandemic’s impact on pesticide availability [32,36], even though this hypothesis should be validated by further studies.

These findings underscore the persistent presence of low-dose pesticide mixtures in feed and food chains and their potential impact on animal, human, and environmental health. Further investigation will clarify the divergent activity of pesticide mixtures and the “cocktail effect” of pesticide absorption [18,19].

Although the detected compound levels are not considered to pose acute risks to cattle and fish according to European guidelines and MRL values, the potential long-term effects of low-dose exposure on animal health and food safety remain unclear, as highlighted by other authors [3,24,45]. Today, as suggested by Palladino et al. in their study, there is a pressing need to update monitoring programs [38]. These programs will lead to a rapid identification of banned or non-target pesticides in animal feed. Indeed, identifying the source of pesticides is a complex issue, as highlighted in several studies [3,24,45]. The potential sources of pesticides may originate from non-compliance with bans, environmental contamination, or drift from neighbouring crops transported by air or other fluids [37,38,46].

So, we invite labs to monitor a wider panel of pesticides with respect to the mandatory ones and compare their findings with our results. This comparison will also be useful for providing an overview that is not influenced by data regionality. Future regulatory practices should build upon these preliminary findings by introducing optional control plans (with a wider pesticide panel list) that align with local authorities and sanitary management. It is crucial that future regulations incorporate more stringent measures. This could involve stricter limits on pesticide use and control, particularly when substances are banned. Additionally, regulations may need to include cross-checking (detecting pesticides in non-target matrices) to detect and address contamination more effectively. Implementing engineering methods to control the spread of pesticides and other harmful substances through the air could also be a key focus. By integrating these practices, future regulatory frameworks can enhance environmental and public health protections, ensuring a more comprehensive approach to managing potential contamination risks.

## Data Availability

Data are contained within the article or Supplementary Material.

## Supplementary Materials

The following supporting information can be downloaded at: https://www.mdpi.com/article/10.3390/toxics12090680/s1, Table S1: Pesticide transition. Pesticide name, parent ion (*m*/*z*), quantitative and qualitative ion (*m*/*z*), collision energy (CE) (V); Table S2: Validated pesticide LOQs; Table S3: Matrix effect; Table S4: Number of samples with detectable residues (n°), mean and deviation standard of the residues detected in the animal-origin- and cereal-based feedstuffs by year of observation (ND: not detected). The DS reported is calculated among all values detected. For measure uncertainty, see SANTE/2019/12682 protocol.

## References

[1] Tudi M., Ruan H.D., Wang L., Lyu J., Sadler R., Connell D., Chu C., Phung D.T. (2021). Agriculture Development, Pesticide Application and Its Impact on the Environment. Int. J. Environ. Res. Public. Health.

[2] Chemical Safety: Pesticides. https://www.who.int/news-room/questions-and-answers/item/chemical-safety-pesticides.

[3] Schleiffer M., Speiser B. (2022). Presence of Pesticides in the Environment, Transition into Organic Food, and Implications for Quality Assurance along the European Organic Food Chain—A Review. Environ. Pollut..

[4] Richardson J.R., Fitsanakis V., Westerink R.H.S., Kanthasamy A.G. (2019). Neurotoxicity of Pesticides. Acta Neuropathol..

[5] Regolamento—396/2005—EN—EUR-Lex. https://eur-lex.europa.eu/legal-content/IT/TXT/?uri=CELEX%3A32005R0396.

[6] EUR-Lex—02002L0032-20191128—EN—EUR-Lex. https://eur-lex.europa.eu/legal-content/EN/TXT/?uri=CELEX%3A02002L0032-20191128.

[7] Maximum Residue Levels—European Commission. https://food.ec.europa.eu/plants/pesticides/maximum-residue-levels_en.

[8] EU Legislation on MRLs—European Commission. https://food.ec.europa.eu/plants/pesticides/maximum-residue-levels/eu-legislation-mrls_en.

[9] Pesticides|EFSA. https://www.efsa.europa.eu/en/topics/topic/pesticides.

[10] Soderlund D.M. (2012). Molecular Mechanisms of Pyrethroid Insecticide Neurotoxicity: Recent Advances. Arch. Toxicol..

[11] Elliott M., Farnham A.W., Janes N.F., Needham P.H., Pulman D.A. (1974). Synthetic Insecticide with a New Order of Activity. Nature.

[12] Verschoyle R.D., Barnes J.M. (1972). Toxicity of Natural and Synthetic Pyrethrins to Rats. Pestic. Biochem. Physiol..

[13] Winter C.K. (2015). Chronic Dietary Exposure to Pesticide Residues in the United States. Int. J. Food Contam..

[14] Hamilton D., Ambrus Á., Dieterle R., Felsot A., Harris C., Petersen B., Racke K., Wong S.S., Gonzalez R., Tanaka K. (2004). Pesticide Residues in Food—Acute Dietary Exposure. Pest. Manag. Sci..

[15] Chiu Y.H., Afeiche M.C., Gaskins A.J., Williams P.L., Petrozza J.C., Tanrikut C., Hauser R., Chavarro J.E. (2015). Fruit and Vegetable Intake and Their Pesticide Residues in Relation to Semen Quality among Men from a Fertility Clinic. Human. Reprod..

[16] Alavanja M.C.R., Hoppin J.A., Kamel F. (2004). Health Effects of Chronic Pesticide Exposure: Cancer and Neurotoxicity. Annu. Rev. Public Health.

[17] Rocheleau C.M., Romitti P.A., Dennis L.K. (2009). Pesticides and Hypospadias: A Meta-Analysis. J. Pediatr. Urol..

[18] Barański M., Średnicka-Tober D., Rempelos L., Hasanaliyeva G., Gromadzka-Ostrowska J., Skwarło-Sońta K., Królikowski T., Rembiałkowska E., Hajslova J., Schulzova V. (2021). Feed Composition Differences Resulting from Organic and Conventional Farming Practices Affect Physiological Parameters in Wistar Rats—Results from a Factorial, Two-Generation Dietary Intervention Trial. Nutrients.

[19] Gómez-Giménez B., Llansola M., Cabrera-Pastor A., Hernández-Rabaza V., Agustí A., Felipo V. (2018). Endosulfan and Cypermethrin Pesticide Mixture Induces Synergistic or Antagonistic Effects on Developmental Exposed Rats Depending on the Analyzed Behavioral or Neurochemical End Points. ACS Chem. Neurosci..

[20] Sherer T.B., Richardson J.R., Testa C.M., Seo B.B., Panov A.V., Yagi T., Matsuno-Yagi A., Miller G.W., Greenamyre J.T. (2007). Mechanism of Toxicity of Pesticides Acting at Complex I: Relevance to Environmental Etiologies of Parkinson’s Disease. J. Neurochem..

[21] Charli A., Jin H., Anantharam V., Kanthasamy A., Kanthasamy A.G. (2016). Alterations in Mitochondrial Dynamics Induced by Tebufenpyrad and Pyridaben in a Dopaminergic Neuronal Cell Culture Model. Neurotoxicology.

[22] Gruetzmacher K., Karesh W.B., Amuasi J.H., Arshad A., Farlow A., Gabrysch S., Jetzkowitz J., Lieberman S., Palmer C., Winkler A.S. (2021). The Berlin Principles on One Health—Bridging Global Health and Conservation. Sci. Total Environ..

[23] Falkenberg T., Ekesi S., Borgemeister C. (2022). Integrated Pest Management (IPM) and One Health—A Call for Action to Integrate. Curr. Opin. Insect Sci..

[24] Penagos-Tabares F., Sulyok M., Faas J., Kriska R., Khiaosa-ard R., Zebeli Q. (2023). Residues of Pesticides and Veterinary Drugs in Diets of Dairy Cattle from Conventional and Organic Farms in Austria. Environ. Pollut..

[25] Silva V., Yang X., Fleskens L., Ritsema C.J., Geissen V. (2022). Environmental and Human Health at Risk—Scenarios to Achieve the Farm to Fork 50% Pesticide Reduction Goals. Environ. Int..

[26] Gilbert G., MacGillivray F.S., Robertson H.L., Jonsson N.N. (2019). Adverse Effects of Routine Bovine Health Treatments Containing Triclabendazole and Synthetic Pyrethroids on the Abundance of Dipteran Larvae in Bovine Faeces. Sci. Rep..

[27] Farm to Fork Strategy—European Commission. https://food.ec.europa.eu/horizontal-topics/farm-fork-strategy_en.

[28] The European Green Deal—European Commission. https://commission.europa.eu/strategy-and-policy/priorities-2019-2024/european-green-deal_en.

[29] (2021). Global Assessment of Soil Pollution: Report. https://openknowledge.fao.org/items/3cba5eed-e9a0-45f0-937b-35f26f2f2723.

[30] Obsolete Pesticides: Contaminated Soil. https://www.fao.org/agriculture/crops/obsolete-pesticides/what-dealing/contaminated/en/.

[31] Ortelli D., Spörri A.S., Edder P. (2018). Veterinary Drug Residue in Food of Animal Origin in Switzerland: A Health Concern?. Chimia.

[32] Carrasco Cabrera L., Di Piazza G., Dujardin B., Medina Pastor P. (2023). The 2021 European Union Report on Pesticide Residues in Food. EFSA J..

[33] Flusso Residuo Prodotti Fitosanitari in RaDiSAN. https://www.salute.gov.it/portale/fitosanitari/dettaglioContenutiFitosanitari.jsp?lingua=italiano&id=5739&area=fitosanitari&menu=flusso.

[34] Andersen G., Poulsen M.E. (2013). Determination of Pesticide Residues in Wheat by GC-MS/MS SweEt Method. https://www.eurl-pesticides.eu/userfiles/file/(11)%20Appendix%203%20Validation%202012%20cerealier%20FC-MSMS%20SweEt%20report%2011.pdf.

[35] Giugliano R., Musolino N., Ciccotelli V., Ferraris C., Savio V., Vivaldi B., Ercolini C., Bianchi D.M., Decastelli L. (2023). Soy, Rice and Oat Drinks: Investigating Chemical and Biological Safety in Plant-Based Milk Alternatives. Nutrients.

[36] Carrasco Cabrera L., Medina Pastor P. (2022). The 2020 European Union Report on Pesticide Residues in Food. EFSA J..

[37] Peterson E.M., Green F.B., Smith P.N. (2020). Pesticides Used on Beef Cattle Feed Yards Are Aerially Transported into the Environment Via Particulate Matter. Environ. Sci. Technol..

[38] Palladino C., Puigvert F., Muela A., Taborda B., Pérez C.A., Pérez-Parada A., Pareja L. (2021). Evaluation of Fusarium Mycotoxins and Fungicide Residues in Barley Grain Produced in Uruguay. J. Agric. Food Res..

[39] Lamichhane J.R., Reay-Jones F.P. (2021). Editorial: Impacts of COVID-19 on Global Plant Health and Crop Protection and the Resulting Effect on Global Food Security and Safety. Crop Prot..

[40] AgroPages-Price & Supply Trend: Insufficient Release of Pesticide Capacity in China after COVID-19 Epidemic-Agricultural News. https://news.agropages.com/News/NewsDetail---34690-e.htm.

[41] Schmidhuber J., Pound J., Qiao B. (2020). COVID-19: Channels of Transmission to Food and Agriculture. https://www.sidalc.net/search/Record/dig-fao-it-20.500.14283-CA8430EN/Description.

[42] Brewin D.G. (2020). The Impact of COVID-19 on the Grains and Oilseeds Sector. Can. J. Agric. Econ. /Rev. Can. D’agroeconomie.

[43] Africa Locust Plague, Coronavirus News: UN Warns of Delays—Bloomberg. https://www.bloomberg.com/news/articles/2020-03-22/coronavirus-slowing-desert-locust-response-in-east-africa.

[44] Regulation—1107/2009—EN—EUR-Lex. https://eur-lex.europa.eu/eli/reg/2009/1107/oj.

[45] Penagos-Tabares F., Sulyok M., Nagl V., Faas J., Krska R., Khiaosa-Ard R., Zebeli Q. (2022). Mixtures of Mycotoxins, Phytoestrogens and Pesticides Co-Occurring in Wet Spent Brewery Grains (BSG) Intended for Dairy Cattle Feeding in Austria. Food Addit. Contam. Part. A Chem. Anal. Control Expo. Risk Assess..

[46] Bonifacio H.F., Maghirang R.G., Trabue S.L., McConnell L.L., Prueger J.H., Bonifacio E.R. (2015). TSP, PM10, and PM2.5 Emissions from a Beef Cattle Feedlot Using the Flux-Gradient Technique. Atmos. Environ..

[47] Guo L., Maghirang R.G., Razote E.B., Trabue S.L., McConnell L.L. (2011). Concentrations of Particulate Matter Emitted from Large Cattle Feedlots in Kansas. J. Air Waste Manag. Assoc..

[48] Gonzales H.B., Maghirang R.G., Wilson J.D., Razote E.B., Guo L. (2011). Measuring Cattle Feedlot Dust Using Laser Diffraction Analysis. Trans. ASABE.

[49] Castro P.M.L., Hayter P.M., Ison A.P., Bull A.T. (1992). Application of a Statistical Design to the Optimization of Culture Medium for Recombinant Interferon-Gamma Production by Chinese Hamster Ovary Cells. Appl. Microbiol. Biotechnol..

[50] Zhao T., Hu K., Li J., Zhu Y., Liu A., Yao K., Liu S. (2021). Current Insights into the Microbial Degradation for Pyrethroids: Strain Safety, Biochemical Pathway, and Genetic Engineering. Chemosphere.

