# Supplementary material for: Monitoring of Non-Maximum-Residue-Level Pesticides in Animal Feed: A Study from 2019 to 2023
Source: Toxics. 2024 Sep 19;12(9):680. doi: 10.3390/toxics12090680 (PMC11435579; doi:10.3390/toxics12090680)
Supplement: Supplementary file 1 [file toxics-12-00680-s001.zip › toxics-3174088-supplementary.pdf]

**Table S1**: Pesticides transition. Pesticides name, parent ion (m/z), quantitative and qualitative ion (m/z), collision energy CE (V).

| Pesticide | Parent Ion (m/z) | Quantitation and Qualification Ions (m/z) | Collision Energy (V) |
|---|---|---|---|
| **Biphenyl** | 154.1 | 152.1 | 25 |
| | 154.1 | 153.1 | 10 |
| **2-Phenylphenol** | 170.1 | 169.1 | 10 |
| | 169.1 | 141.1 | 10 |
| **Diphenylamine** | 168.2 | 167.1 | 15 |
| | 169.2 | 168.2 | 10 |
| **Trifluralin** | 306.1 | 264.1 | 5 |
| | 306.1 | 206.1 | 10 |
| **Fenthion** | 278 | 109.1 | 15 |
| | 278 | 169.1 | 15 |
| **Cyprodinil** | 224.1 | 208.2 | 15 |
| | 225.2 | 210.2 | 15 |
| **Pendimethalin** | 252.1 | 162.2 | 10 |
| | 252.1 | 161.2 | 15 |
| **TPP (SI)** | 325 | 169.1 | 15 |
| | 326.1 | 233.1 | 10 |
| **Piperonyl butoxide** | 176.1 | 103.1 | 10 |
| | 176.1 | 131.1 | 20 |
| **Tetramethrin** | 164.1 | 107.1 | 10 |
| | 164.1 | 77.1 | 25 |
| **Difenoconazole** | 323 | 265.1 | 10 |
| | 265 | 139.1 | 35 |
| **Desmedipham** | 181.1 | 109.1 | 10 |
| | 109.1 | 8.1 | 10 |
| **Pyrimethanil** | 198.1 | 118.1 | 30 |
| | 198.1 | 182.1 | 20 |
| **Chlorpyrifos-Methyl** | 286,0 | 93.1 | 20 |
| | 286 | 271 | 10 |
| **β- Endosulfan** | 241 | 206 | 10 |
| | 207 | 172.1 | 10 |
| **Malaoxon** | 127 | 99.1 | 5 |
| | 127 | 109.1 | 10 |
| **Fipronil** | 366.9 | 213 | 25 |
| | 369 | 215 | 25 |
| **Procymidone** | 283 | 96.1 | 10 |
| | 285 | 96.1 | 10 |
| **Triadimenol** | 128 | 65.1 | 20 |
| | 168.1 | 70.1 | 10 |
| **Fludioxonil** | 154.1 | 127.1 | 25 |
| | 248 | 127.1 | 10 |
| **Fenazaquin** | 160.2 | 117.1 | 20 |
| | 145.1 | 91.1 | 25 |
| **Phosalone** | 182 | 75.1 | 30 |
| | 182 | 111.1 | 15 |
| **λ-Cyhalothrin** | 181.1 | 152.1 | 20 |
| | 197 | 141.1 | 10 |

| | | | |
|---|---|---|---|
| **Deltamethrin** | 252.9 | 172.1 | 5 |
| | 252.9 | 93.1 | 15 |
| **Azoxystrobin** | 344.1 | 329.2 | 10 |
| | 388.1 | 360.2 | 10 |
| **Pirimiphos-methyl** | 290.1 | 125.1 | 20 |
| | 305.1 | 180.2 | 5 |
| **Malathion** | 173.1 | 127.1 | 5 |
| | 173.1 | 99.1 | 10 |
| **Chlorpyrifos** | 314 | 258 | 10 |
| | 316 | 260 | 10 |
| **Profenofos** | 339 | 269 | 10 |
| | 337 | 267 | 10 |
| **Dieldrin** | 276.9 | 241 | 5 |
| | 276.9 | 207.1 | 20 |
| **Fipronil-sulfone** | 383 | 255.1 | 15 |
| | 385 | 257 | 15 |
| **p,p'-DDD + o,p'-DDT** | 235 | 165.1 | 20 |
| | 237 | 165.1 | 20 |
| **o,p'-DDT** | 235 | 165.1 | 20 |
| | 237 | 165.1 | 20 |
| **p,p'- DDE** | 246.0 | 176.1 | 30 |
| | 371.9 | 248.1 | 15 |
| **Permethrin** | 183.1 | 168.1 | 10 |
| | 183.1 | 153.1 | 10 |
| **Boscalid** | 140 | 112 | 10 |
| | 140 | 76.1 | 20 |
| **CyPermethrin** | 163 | 127.1 | 5 |
| | 163 | 91.1 | 10 |
| **Bixafen** | 413 | 159.1 | 10 |
| | 159 | 139.1 | 10 |
| **Etofenprox** | 163.1 | 135.1 | 10 |
| | 163.1 | 107.1 | 15 |
| **Fenvalerate** | 125 | 89.1 | 20 |
| | 167.1 | 125.1 | 10 |
| **τ-Fluvalinate** | 250.1 | 200.1 | 15 |
| | 250.1 | 55.1 | 15 |

**Table S2**: Validated pesticides LOQs.

| Analita | LOQ |
|---|---|
| p,p'-DDD + o,p'-DDT | 0.004 |
| Chlorpyrifos, cis-Chlordane, Cyprodinil, Dieldrin, Endrin, Endrin-ketone, Fenazaquin, Fenitrothion, Fenpropimorph, Fenthion, Heptachlor-exo-epoxide, Malaoxon, Malathion, Mepanipyrim, Metalaxyl, Metolachlor, Metribuzin, Paraoxon-methyl, Pendimethalin, Permethrin, Pirimiphos, Pirimiphos-methyl, Pendimethalin, Permethrin, Pirimiphos, Pirimiphos-methyl, Propargite, Pyridaben, trans-Chlordane, Vinclozolin | 0.005 |
| All other pesticides | 0.002 |

**Table S3**: Matrix effect.

| Pesticide | Slope Solvent | Slope wheat-based feed | Matrix effect wheat-based feed | Slope corn-based feed | Matrix effect corn-based feed | Slope animal-origin based feed | Matrix effect animal-origin based feed |
|---|---|---|---|---|---|---|---|
| **2-Phenylphenol** | 0.09 | 0.08 | Medium | 0.08 | Not Significant | 0.08 | Not Significant |
| **Azoxystrobin** | 0.00 | 0.02 | Medium | 0.02 | Strong | 0.01 | Strong |
| **Biphenyl** | 0.11 | 0.08 | Medium | 0.09 | Not Significant | 0.09 | Not Significant |
| **Bixafen** | 0.03 | 0.04 | Not Significant | 0.03 | Not Significant | 0.03 | Not Significant |
| **Boscalid** | 0.12 | 0.12 | Medium | 0.11 | Not Significant | 0.09 | Medium |
| **Chlorpyrifos** | 0.02 | 0.02 | Not Significant | 0.02 | Not Significant | 0.02 | Not Significant |
| **Chlorpyrifos-methyl** | 0.02 | 0.01 | Not Significant | 0.02 | Not Significant | 0.02 | Not Significant |
| **Cypermethrin** | 0.04 | 0.05 | Not Significant | 0.04 | Not Significant | 0.04 | Not Significant |
| **Cyprodinil** | 0.04 | 0.04 | Not Significant | 0.04 | Not Significant | 0.04 | Not Significant |
| **Deltamethrin** | 0.03 | 0.03 | Not Significant | 0.03 | Not Significant | 0.03 | Not Significant |
| **Desmedipham** | 0.01 | 0.01 | Not Significant | 0.01 | Not Significant | 0.00 | Strong |
| **Dieldrin** | 0.00 | 0.00 | Not Significant | 0.00 | Medium | 0.04 | Strong |
| **Difenoconazole** | 0.05 | 0.06 | Medium | 0.05 | Not Significant | 0.04 | Not Significant |
| **Diphenylamine** | 0.11 | 0.09 | Medium | 0.10 | Not Significant | 0.10 | Not Significant |
| **Etofenprox** | 0.21 | 0.21 | Not Significant | 0.21 | Not Significant | 0.15 | Medium |
| **Fenazaquin** | 0.00 | 0.03 | Not Significant | 0.04 | Strong | 0.03 | Strong |
| **Fenthion** | 0.02 | 0.02 | Medium | 0.01 | Not Significant | 0.02 | Not Significant |
| **Fenvalerate** | 0.01 | 0.01 | Not Significant | 0.01 | Not Significant | 0.01 | Medium |
| **Fipronil** | 0.02 | 0.02 | Not Significant | 0.02 | Not Significant | 0.02 | Not Significant |
| **Fipronil-sulfone** | 0.03 | 0.03 | Not Significant | 0.03 | Not Significant | 0.03 | Not Significant |
| **Fludioxonil** | 0.06 | 0.04 | Medium | 0.03 | Strong | 0.06 | Not Significant |
| **Malaoxon** | 0.02 | 0.04 | Not Significant | 0.04 | Strong | 0.04 | Strong |
| **Malathion** | 0.04 | 0.03 | Medium | 0.03 | Not Significant | 0.03 | Not Significant |
| **p,p'-DDD + o,p'-DDT** | 0.12 | 0.12 | Not Significant | 0.11 | Not Significant | 0.06 | Strong |
| **p,p'-DDE** | 0.06 | 0.05 | Not Significant | 0.02 | Strong | 0.06 | Not Significant |
| **p,p'-DDT** | 0.03 | 0.03 | Medium | 0.02 | Medium | 0.83 | Strong |
| **Pendimethalin** | 0.02 | 0.02 | Not Significant | 0.02 | Not Significant | 0.02 | Not Significant |
| **Permethrin** | 0.03 | 0.03 | Not Significant | 0.03 | Not Significant | 0.02 | Medium |
| **Phosalone** | 0.02 | 0.03 | Not Significant | 0.03 | Not Significant | 0.02 | Not Significant |
| **Piperonyl-butoxide** | 0.05 | 0.05 | Not Significant | 0.05 | Not Significant | 0.04 | Not Significant |
| **Pirimiphos-methyl** | 0.02 | 0.02 | Not Significant | 0.02 | Not Significant | 0.02 | Medium |
| **Procymidone** | 0.02 | 0.01 | Medium | 0.01 | Medium | 0.01 | Strong |
| **Profenofos** | 0.02 | 0.02 | Not Significant | 0.01 | Not Significant | 0.02 | Medium |
| **Pyrimethanil** | 0.02 | 0.02 | Medium | 0.02 | Not Significant | 0.02 | Not Significant |
| **Tetramethrin** | 0.06 | 0.06 | Not Significant | 0.07 | Not Significant | 0.06 | Not Significant |
| **Triadimenol** | 0.00 | 0.01 | Medium | 0.01 | Not Significant | 0.00 | Not Significant |
| **Trifluralin** | 0.05 | 0.04 | Not Significant | 0.04 | Not Significant | 0.06 | Not Significant |
| **β-Endosulfan** | 0.00 | 0.00 | Not Significant | 0.00 | Not Significant | 0.00 | Medium |
| **λ-Cyhalothrin** | 0.04 | 0.04 | Not Significant | 0.04 | Not Significant | 0.03 | Not Significant |
| **τ-Fluvalinate** | 0.02 | 0.02 | Not Significant | 0.02 | Not Significant | 0.01 | Medium |

**Table S4**: Number of the samples with detectable residues (n°), mean and deviation standard of the residues detected in the animal origin and cereals-based feedstuffs, by years of observation (ND: not detected). The DS reported is calculated among all values detected. For the measure uncertainty see SANTE/2019/12682 protocol.

| Compound | Feed | Year | n° | Concentration (ppm) | |
|---|---|---|---|---|---|
| | | | | Mean | DS |
| **2-Phenylphenol** | Animal-origin based | 2019 | 0 | < LOQ | / |
| **2-Phenylphenol** | Animal-origin based | 2020 | 5 | 0.03 | 0.02 |
| **2-Phenylphenol** | Animal-origin based | 2021 | 7 | 0.02 | 0.02 |
| **2-Phenylphenol** | Animal-origin based | 2022 | 4 | 0.02 | 0.01 |
| **2-Phenylphenol** | Animal-origin based | 2023 | 4 | 0.01 | 0.01 |
| **2-Phenylphenol** | Cereal-based | 2021 | 0 | < LOQ | / |
| **2-Phenylphenol** | Cereal-based | 2022 | 0 | < LOQ | / |
| **2-Phenylphenol** | Cereal-based | 2023 | 0 | < LOQ | / |
| **Azoxystrobin** | Animal-origin based | 2019 | 0 | < LOQ | / |
| **Azoxystrobin** | Animal-origin based | 2020 | 0 | < LOQ | / |
| **Azoxystrobin** | Animal-origin based | 2021 | 1 | < LOQ | / |
| **Azoxystrobin** | Cereal-based | 2021 | 1 | 0.01 | / |
| **Azoxystrobin** | Animal-origin based | 2022 | 0 | < LOQ | / |
| **Azoxystrobin** | Cereal-based | 2022 | 1 | 0.96 | / |
| **Azoxystrobin** | Animal-origin based | 2023 | 0 | < LOQ | / |
| **Azoxystrobin** | Cereal-based | 2023 | 5 | 0.01 | / |
| **Beta-Endosulfan** | Animal-origin based | 2019 | 0 | < LOQ | / |
| **Beta-Endosulfan** | Animal-origin based | 2020 | 1 | 0.03 | / |
| **Beta-Endosulfan** | Animal-origin based | 2021 | 0 | < LOQ | / |
| **Beta-Endosulfan** | Cereal-based | 2021 | 0 | < LOQ | / |
| **Beta-Endosulfan** | Animal-origin based | 2022 | 0 | < LOQ | / |
| **Beta-Endosulfan** | Cereal-based | 2022 | 0 | < LOQ | / |
| **Beta-Endosulfan** | Animal-origin based | 2023 | 0 | < LOQ | / |
| **Beta-Endosulfan** | Cereal-based | 2023 | 0 | < LOQ | / |
| **Biphenyl** | Cereal-based | 2021 | 0 | < LOQ | / |
| **Biphenyl** | Cereal-based | 2022 | 1 | 0.01 | / |
| **Biphenyl** | Cereal-based | 2023 | 1 | 0.01 | / |
| **Biphenyl** | Animal-origin based | 2019 | 0 | < LOQ | / |
| **Biphenyl** | Animal-origin based | 2020 | 0 | < LOQ | / |
| **Biphenyl** | Animal-origin based | 2021 | 0 | < LOQ | / |
| **Biphenyl** | Animal-origin based | 2022 | 0 | < LOQ | / |
| **Biphenyl** | Animal-origin based | 2023 | 0 | < LOQ | / |
| **Bixafen** | Animal-origin based | 2019 | 0 | < LOQ | / |
| **Bixafen** | Animal-origin based | 2020 | 0 | < LOQ | / |
| **Bixafen** | Animal-origin based | 2021 | 0 | < LOQ | / |
| **Bixafen** | Cereal-based | 2021 | 0 | < LOQ | / |
| **Bixafen** | Animal-origin based | 2022 | 0 | < LOQ | / |
| **Bixafen** | Cereal-based | 2022 | 0 | < LOQ | / |
| **Bixafen** | Animal-origin based | 2023 | 1 | 0.04 | / |
| **Bixafen** | Cereal-based | 2023 | 0 | < LOQ | / |
| **Boscalid** | Animal-origin based | 2019 | 0 | < LOQ | / |

| | | | | | |
|---|---|---|---|---|---|
| **Boscalid** | Animal-origin based | 2020 | 2 | < LOQ | / |
| **Boscalid** | Animal-origin based | 2021 | 6 | 0.01 | 0.01 |
| **Boscalid** | Cereal-based | 2021 | 0 | < LOQ | / |
| **Boscalid** | Animal-origin based | 2022 | 0 | < LOQ | / |
| **Boscalid** | Cereal-based | 2022 | 0 | < LOQ | / |
| **Boscalid** | Animal-origin based | 2023 | 1 | 0.01 | / |
| **Boscalid** | Cereal-based | 2023 | 2 | 0.01 | 0.01 |
| **Chlorpyrifos** | Animal-origin based | 2019 | 1 | < LOQ | / |
| **Chlorpyrifos** | Animal-origin based | 2020 | 0 | < LOQ | / |
| **Chlorpyrifos** | Animal-origin based | 2021 | 0 | < LOQ | / |
| **Chlorpyrifos** | Cereal-based | 2021 | 0 | < LOQ | / |
| **Chlorpyrifos** | Animal-origin based | 2022 | 0 | < LOQ | / |
| **Chlorpyrifos** | Cereal-based | 2022 | 1 | 0.01 | / |
| **Chlorpyrifos** | Animal-origin based | 2023 | 0 | < LOQ | / |
| **Chlorpyrifos** | Cereal-based | 2023 | 1 | 0.01 | / |
| **Chlorpyrifos-methyl** | Cereal-based | 2021 | 0 | < LOQ | / |
| **Chlorpyrifos-methyl** | Cereal-based | 2022 | 0 | < LOQ | / |
| **Chlorpyrifos-methyl** | Cereal-based | 2023 | 1 | < LOQ | / |
| **Chlorpyrifos-methyl** | Animal-origin based | 2019 | 3 | 0.02 | 0.02 |
| **Chlorpyrifos-methyl** | Animal-origin based | 2020 | 0 | < LOQ | / |
| **Chlorpyrifos-methyl** | Animal-origin based | 2021 | 0 | < LOQ | / |
| **Chlorpyrifos-methyl** | Animal-origin based | 2022 | 0 | < LOQ | / |
| **Chlorpyrifos-methyl** | Animal-origin based | 2023 | 0 | < LOQ | / |
| **Cypermethrin** | Animal-origin based | 2019 | 6 | 0.02 | 0.02 |
| **Cypermethrin** | Animal-origin based | 2020 | 5 | 0.02 | 0.03 |
| **Cypermethrin** | Animal-origin based | 2021 | 5 | 0.01 | 0.01 |
| **Cypermethrin** | Animal-origin based | 2022 | 4 | < LOQ | / |
| **Cypermethrin** | Animal-origin based | 2023 | 5 | 0.02 | 0.04 |
| **Cypermethrin** | Cereal-based | 2021 | 5 | 0.04 | 0.02 |
| **Cypermethrin** | Cereal-based | 2022 | 2 | 0.01 | 0.01 |
| **Cypermethrin** | Cereal-based | 2023 | 10 | 0.03 | 0.04 |
| **Cyprodinil** | Animal-origin based | 2019 | 0 | < LOQ | / |
| **Cyprodinil** | Animal-origin based | 2020 | 0 | < LOQ | / |
| **Cyprodinil** | Animal-origin based | 2021 | 0 | < LOQ | / |
| **Cyprodinil** | Cereal-based | 2021 | 0 | < LOQ | / |
| **Cyprodinil** | Animal-origin based | 2022 | 0 | < LOQ | / |
| **Cyprodinil** | Cereal-based | 2022 | 1 | < LOQ | / |
| **Cyprodinil** | Animal-origin based | 2023 | 0 | < LOQ | / |
| **Cyprodinil** | Cereal-based | 2023 | 1 | 0.01 | / |
| **Deltamethrin** | Animal-origin based | 2019 | 11 | 0.03 | 0.03 |
| **Deltamethrin** | Animal-origin based | 2020 | 7 | 0.01 | 0.01 |
| **Deltamethrin** | Animal-origin based | 2021 | 7 | 0.01 | 0.01 |
| **Deltamethrin** | Animal-origin based | 2022 | 8 | 0.03 | 0.04 |
| **Deltamethrin** | Animal-origin based | 2023 | 4 | 0.05 | 0.03 |
| **Deltamethrin** | Cereal-based | 2021 | 6 | 0.02 | 0.02 |
| **Deltamethrin** | Cereal-based | 2022 | 7 | 0.01 | 0.01 |
| **Deltamethrin** | Cereal-based | 2023 | 17 | 0.12 | 0.32 |

| | | | | | |
|---|---|---|---|---|---|
| **Desmedipham** | Animal-origin based | 2019 | 0 | < LOQ | / |
| **Desmedipham** | Animal-origin based | 2020 | 1 | 0.01 | / |
| **Desmedipham** | Animal-origin based | 2021 | 0 | < LOQ | / |
| **Desmedipham** | Cereal-based | 2021 | 1 | 0.02 | / |
| **Desmedipham** | Animal-origin based | 2022 | 0 | < LOQ | / |
| **Desmedipham** | Cereal-based | 2022 | 0 | < LOQ | / |
| **Desmedipham** | Animal-origin based | 2023 | 0 | < LOQ | / |
| **Desmedipham** | Cereal-based | 2023 | 0 | < LOQ | / |
| **Difenoconazole** | Animal-origin based | 2019 | 0 | < LOQ | / |
| **Difenoconazole** | Animal-origin based | 2020 | 0 | < LOQ | / |
| **Difenoconazole** | Animal-origin based | 2021 | 0 | < LOQ | / |
| **Difenoconazole** | Cereal-based | 2021 | 0 | < LOQ | / |
| **Difenoconazole** | Animal-origin based | 2022 | 1 | 0.03 | / |
| **Difenoconazole** | Cereal-based | 2022 | 1 | 0.03 | / |
| **Difenoconazole** | Animal-origin based | 2023 | 0 | < LOQ | / |
| **Difenoconazole** | Cereal-based | 2023 | 2 | 0.01 | / |
| **Diphenylamine** | Cereal-based | 2021 | 0 | < LOQ | / |
| **Diphenylamine** | Cereal-based | 2022 | 0 | < LOQ | / |
| **Diphenylamine** | Cereal-based | 2023 | 0 | < LOQ | / |
| **Diphenylamine** | Animal-origin based | 2019 | 0 | < LOQ | / |
| **Diphenylamine** | Animal-origin based | 2020 | 0 | < LOQ | / |
| **Diphenylamine** | Animal-origin based | 2021 | 0 | < LOQ | / |
| **Diphenylamine** | Animal-origin based | 2022 | 1 | < LOQ | / |
| **Diphenylamine** | Animal-origin based | 2023 | 0 | < LOQ | / |
| **Etofenprox** | Animal-origin based | 2019 | 0 | < LOQ | / |
| **Etofenprox** | Animal-origin based | 2020 | 0 | < LOQ | / |
| **Etofenprox** | Animal-origin based | 2021 | 0 | < LOQ | / |
| **Etofenprox** | Cereal-based | 2021 | 0 | < LOQ | / |
| **Etofenprox** | Animal-origin based | 2022 | 0 | < LOQ | / |
| **Etofenprox** | Cereal-based | 2022 | 0 | < LOQ | / |
| **Etofenprox** | Animal-origin based | 2023 | 0 | < LOQ | / |
| **Etofenprox** | Cereal-based | 2023 | 3 | 0.26 | 0.35 |
| **Fenazaquin** | Animal-origin based | 2019 | 0 | < LOQ | / |
| **Fenazaquin** | Animal-origin based | 2020 | 1 | 0.01 | / |
| **Fenazaquin** | Animal-origin based | 2021 | 0 | < LOQ | / |
| **Fenazaquin** | Cereal-based | 2021 | 0 | < LOQ | / |
| **Fenazaquin** | Animal-origin based | 2022 | 1 | 0.01 | / |
| **Fenazaquin** | Cereal-based | 2022 | 0 | < LOQ | / |
| **Fenazaquin** | Animal-origin based | 2023 | 0 | < LOQ | / |
| **Fenazaquin** | Cereal-based | 2023 | 0 | < LOQ | / |
| **Fenthion** | Animal-origin based | 2019 | 0 | < LOQ | / |
| **Fenthion** | Animal-origin based | 2020 | 0 | < LOQ | / |
| **Fenthion** | Animal-origin based | 2021 | 0 | < LOQ | / |
| **Fenthion** | Cereal-based | 2021 | 0 | < LOQ | / |
| **Fenthion** | Animal-origin based | 2022 | 0 | < LOQ | / |
| **Fenthion** | Cereal-based | 2022 | 0 | < LOQ | / |
| **Fenthion** | Animal-origin based | 2023 | 1 | 0.01 | / |

| | | | | | |
|---|---|---|---|---|---|
| **Fenthion** | Cereal-based | 2023 | 0 | < LOQ | / |
| **Fenvalerate** | Animal-origin based | 2019 | 0 | < LOQ | / |
| **Fenvalerate** | Animal-origin based | 2020 | 0 | < LOQ | / |
| **Fenvalerate** | Animal-origin based | 2021 | 0 | < LOQ | / |
| **Fenvalerate** | Cereal-based | 2021 | 0 | < LOQ | / |
| **Fenvalerate** | Animal-origin based | 2022 | 0 | < LOQ | / |
| **Fenvalerate** | Cereal-based | 2022 | 0 | < LOQ | / |
| **Fenvalerate** | Animal-origin based | 2023 | 1 | 0.32 | / |
| **Fenvalerate** | Cereal-based | 2023 | 1 | 0.01 | / |
| **Fipronil** | Animal-origin based | 2019 | 0 | < LOQ | / |
| **Fipronil** | Animal-origin based | 2020 | 0 | < LOQ | / |
| **Fipronil** | Animal-origin based | 2021 | 0 | < LOQ | / |
| **Fipronil** | Cereal-based | 2021 | 0 | < LOQ | / |
| **Fipronil** | Animal-origin based | 2022 | 0 | < LOQ | / |
| **Fipronil** | Cereal-based | 2022 | 0 | < LOQ | / |
| **Fipronil** | Animal-origin based | 2023 | 0 | < LOQ | / |
| **Fipronil** | Cereal-based | 2023 | 1 | 0.01 | / |
| **Fludioxonil** | Animal-origin based | 2019 | 0 | < LOQ | / |
| **Fludioxonil** | Animal-origin based | 2020 | 0 | < LOQ | / |
| **Fludioxonil** | Animal-origin based | 2021 | 0 | < LOQ | / |
| **Fludioxonil** | Cereal-based | 2021 | 0 | < LOQ | / |
| **Fludioxonil** | Animal-origin based | 2022 | 1 | < LOQ | / |
| **Fludioxonil** | Cereal-based | 2022 | 1 | 0.01 | / |
| **Fludioxonil** | Animal-origin based | 2023 | 1 | < LOQ | / |
| **Fludioxonil** | Cereal-based | 2023 | 0 | < LOQ | / |
| **Lambda-cyhalothrin** | Cereal-based | 2021 | 0 | < LOQ | / |
| **Lambda-cyhalothrin** | Cereal-based | 2022 | 1 | 0.34 | / |
| **Lambda-cyhalothrin** | Cereal-based | 2023 | 1 | 0.02 | / |
| **Lambda-cyhalothrin** | Animal-origin based | 2019 | 0 | < LOQ | / |
| **Lambda-cyhalothrin** | Animal-origin based | 2020 | 0 | < LOQ | / |
| **Lambda-cyhalothrin** | Animal-origin based | 2021 | 0 | < LOQ | / |
| **Lambda-cyhalothrin** | Animal-origin based | 2022 | 4 | 0.02 | 0.01 |
| **Lambda-cyhalothrin** | Animal-origin based | 2023 | 0 | < LOQ | / |
| **Malaoxon** | Animal-origin based | 2019 | 2 | 0.01 | / |
| **Malaoxon** | Animal-origin based | 2020 | 3 | 0.02 | 0.03 |
| **Malaoxon** | Animal-origin based | 2021 | 0 | < LOQ | / |
| **Malaoxon** | Cereal-based | 2021 | 0 | < LOQ | / |
| **Malaoxon** | Animal-origin based | 2022 | 0 | < LOQ | / |
| **Malaoxon** | Cereal-based | 2022 | 0 | < LOQ | / |
| **Malaoxon** | Animal-origin based | 2023 | 0 | < LOQ | / |
| **Malaoxon** | Cereal-based | 2023 | 0 | < LOQ | / |
| **Malathion** | Animal-origin based | 2019 | 0 | < LOQ | / |
| **Malathion** | Animal-origin based | 2020 | 1 | 0.01 | / |
| **Malathion** | Animal-origin based | 2021 | 0 | < LOQ | / |
| **Malathion** | Cereal-based | 2021 | 0 | < LOQ | / |
| **Malathion** | Animal-origin based | 2022 | 0 | < LOQ | / |
| **Malathion** | Cereal-based | 2022 | 0 | < LOQ | / |

| | | | | | |
|---|---|---|---|---|---|
| **Malathion** | Animal-origin based | 2023 | 0 | < LOQ | / |
| **Malathion** | Cereal-based | 2023 | 0 | < LOQ | / |
| **o, p'-DDT** | Cereal-based | 2021 | 0 | < LOQ | / |
| **o, p'-DDT** | Cereal-based | 2022 | 0 | < LOQ | / |
| **o, p'-DDT** | Cereal-based | 2023 | 0 | < LOQ | / |
| **o, p'-DDT** | Animal-origin based | 2019 | 1 | < LOQ | / |
| **o, p'-DDT** | Animal-origin based | 2020 | 0 | < LOQ | / |
| **o, p'-DDT** | Animal-origin based | 2021 | 0 | < LOQ | / |
| **o, p'-DDT** | Animal-origin based | 2022 | 0 | < LOQ | / |
| **o, p'-DDT** | Animal-origin based | 2023 | 0 | < LOQ | / |
| **p, p'-DDE** | Cereal-based | 2021 | 0 | < LOQ | / |
| **p, p'-DDE** | Cereal-based | 2022 | 0 | < LOQ | / |
| **p, p'-DDE** | Cereal-based | 2023 | 0 | < LOQ | / |
| **p, p'-DDE** | Animal-origin based | 2019 | 1 | < LOQ | / |
| **p, p'-DDE** | Animal-origin based | 2020 | 0 | < LOQ | / |
| **p, p'-DDE** | Animal-origin based | 2021 | 1 | 0.01 | / |
| **p, p'-DDE** | Animal-origin based | 2022 | 0 | < LOQ | / |
| **p, p'-DDE** | Animal-origin based | 2023 | 0 | < LOQ | / |
| **Pendimethalin** | Cereal-based | 2021 | 1 | 0.01 | / |
| **Pendimethalin** | Cereal-based | 2022 | 0 | < LOQ | / |
| **Pendimethalin** | Cereal-based | 2023 | 0 | < LOQ | / |
| **Pendimethalin** | Animal-origin based | 2019 | 0 | < LOQ | / |
| **Pendimethalin** | Animal-origin based | 2020 | 0 | < LOQ | / |
| **Pendimethalin** | Animal-origin based | 2021 | 0 | < LOQ | / |
| **Pendimethalin** | Animal-origin based | 2022 | 0 | < LOQ | / |
| **Pendimethalin** | Animal-origin based | 2023 | 0 | < LOQ | / |
| **Permethrin** | Animal-origin based | 2019 | 2 | 0.02 | 0.01 |
| **Permethrin** | Animal-origin based | 2020 | 1 | 0.01 | / |
| **Permethrin** | Animal-origin based | 2021 | 5 | 0.01 | 0.01 |
| **Permethrin** | Animal-origin based | 2022 | 1 | 0.01 | / |
| **Permethrin** | Animal-origin based | 2023 | 5 | 0.16 | 0.14 |
| **Permethrin** | Cereal-based | 2021 | 0 | < LOQ | / |
| **Permethrin** | Cereal-based | 2022 | 0 | < LOQ | / |
| **Permethrin** | Cereal-based | 2023 | 2 | 0.05 | 0.03 |
| **Phosalone** | Animal-origin based | 2019 | 0 | < LOQ | / |
| **Phosalone** | Animal-origin based | 2020 | 0 | < LOQ | / |
| **Phosalone** | Animal-origin based | 2021 | 0 | < LOQ | / |
| **Phosalone** | Cereal-based | 2021 | 0 | < LOQ | / |
| **Phosalone** | Animal-origin based | 2022 | 0 | < LOQ | / |
| **Phosalone** | Cereal-based | 2022 | 0 | < LOQ | / |
| **Phosalone** | Animal-origin based | 2023 | 0 | < LOQ | / |
| **Phosalone** | Cereal-based | 2023 | 1 | 0.02 | / |
| **Piperonyl butoxide** | Animal-origin based | 2019 | 8 | 0.29 | 0.34 |
| **Piperonyl butoxide** | Animal-origin based | 2020 | 9 | 0.10 | 0.11 |
| **Piperonyl butoxide** | Animal-origin based | 2021 | 13 | 0.07 | 0.08 |
| **Piperonyl butoxide** | Animal-origin based | 2022 | 13 | 0.15 | 0.20 |
| **Piperonyl butoxide** | Animal-origin based | 2023 | 8 | 0.16 | 0.18 |

| | | | | | |
|---|---|---|---|---|---|
| **Piperonyl butoxide** | Cereal-based | 2021 | 19 | 0.20 | 0.37 |
| **Piperonyl butoxide** | Cereal-based | 2022 | 10 | 0.14 | 0.14 |
| **Piperonyl butoxide** | Cereal-based | 2023 | 28 | 0.24 | 0.38 |
| **Pirimiphos-methyl** | Animal-origin based | 2019 | 12 | 0.07 | 0.07 |
| **Pirimiphos-methyl** | Animal-origin based | 2020 | 9 | 0.03 | 0.04 |
| **Pirimiphos-methyl** | Animal-origin based | 2021 | 10 | 0.05 | 0.13 |
| **Pirimiphos-methyl** | Cereal-based | 2021 | 13 | 0.08 | 0.06 |
| **Pirimiphos-methyl** | Animal-origin based | 2022 | 10 | 0.01 | 0.01 |
| **Pirimiphos-methyl** | Cereal-based | 2022 | 5 | 0.14 | 0.21 |
| **Pirimiphos-methyl** | Animal-origin based | 2023 | 7 | 0.01 | / |
| **Pirimiphos-methyl** | Cereal-based | 2023 | 16 | 0.08 | 0.06 |
| **p, p'-DDD+ o, p' - DDT** | Cereal-based | 2021 | 0 | < LOQ | / |
| **p, p'-DDD+ o, p' - DDT** | Cereal-based | 2022 | 0 | < LOQ | / |
| **p, p'-DDD+ o, p' - DDT** | Cereal-based | 2023 | 0 | < LOQ | / |
| **p, p'-DDD+ o, p' - DDT** | Animal-origin based | 2019 | 1 | < LOQ | / |
| **p, p'-DDD+ o, p' - DDT** | Animal-origin based | 2020 | 0 | < LOQ | / |
| **p, p'-DDD+ o, p' - DDT** | Animal-origin based | 2021 | 0 | < LOQ | / |
| **p, p'-DDD+ o, p' - DDT** | Animal-origin based | 2022 | 0 | < LOQ | / |
| **p, p'-DDD+ o, p' - DDT** | Animal-origin based | 2023 | 0 | < LOQ | / |
| **Procymidone** | Animal-origin based | 2019 | 0 | < LOQ | / |
| **Procymidone** | Animal-origin based | 2020 | 1 | 0.03 | / |
| **Procymidone** | Animal-origin based | 2021 | 0 | < LOQ | / |
| **Procymidone** | Cereal-based | 2021 | 0 | < LOQ | / |
| **Procymidone** | Animal-origin based | 2022 | 0 | < LOQ | / |
| **Procymidone** | Cereal-based | 2022 | 0 | < LOQ | / |
| **Procymidone** | Animal-origin based | 2023 | 0 | < LOQ | / |
| **Procymidone** | Cereal-based | 2023 | 0 | < LOQ | / |
| **Pyridaben** | Animal-origin based | 2019 | 5 | 0.01 | / |
| **Pyridaben** | Animal-origin based | 2020 | 0 | < LOQ | / |
| **Pyridaben** | Animal-origin based | 2021 | 0 | < LOQ | / |
| **Pyridaben** | Cereal-based | 2021 | 0 | < LOQ | / |
| **Pyridaben** | Animal-origin based | 2022 | 1 | 0.01 | / |
| **Pyridaben** | Cereal-based | 2022 | 1 | 0.03 | / |
| **Pyridaben** | Animal-origin based | 2023 | 0 | < LOQ | / |
| **Pyridaben** | Cereal-based | 2023 | 0 | < LOQ | / |
| **Pyrimethanil** | Animal-origin based | 2019 | 0 | < LOQ | / |
| **Pyrimethanil** | Animal-origin based | 2020 | 0 | < LOQ | / |
| **Pyrimethanil** | Animal-origin based | 2021 | 0 | < LOQ | / |
| **Pyrimethanil** | Cereal-based | 2021 | 0 | < LOQ | / |
| **Pyrimethanil** | Animal-origin based | 2022 | 0 | < LOQ | / |
| **Pyrimethanil** | Cereal-based | 2022 | 0 | < LOQ | / |
| **Pyrimethanil** | Animal-origin based | 2023 | 0 | < LOQ | / |
| **Pyrimethanil** | Cereal-based | 2023 | 1 | 0.83 | / |
| **tau-Fluvalinate** | Animal-origin based | 2019 | 0 | < LOQ | / |
| **tau-Fluvalinate** | Animal-origin based | 2020 | 0 | < LOQ | / |
| **tau-Fluvalinate** | Animal-origin based | 2021 | 0 | < LOQ | / |
| **tau-Fluvalinate** | Cereal-based | 2021 | 0 | < LOQ | / |

| | | | | | |
|---|---|---|---|---|---|
| **tau-Fluvalinate** | Animal-origin based | 2022 | 0 | < LOQ | / |
| **tau-Fluvalinate** | Cereal-based | 2022 | 0 | < LOQ | / |
| **tau-Fluvalinate** | Animal-origin based | 2023 | 0 | < LOQ | / |
| **tau-Fluvalinate** | Cereal-based | 2023 | 4 | 0.07 | 0.11 |
| **Tetramethrin** | Animal-origin based | 2019 | 0 | < LOQ | / |
| **Tetramethrin** | Animal-origin based | 2020 | 0 | < LOQ | / |
| **Tetramethrin** | Animal-origin based | 2021 | 1 | 0.02 | / |
| **Tetramethrin** | Cereal-based | 2021 | 2 | 0.08 | / |
| **Tetramethrin** | Animal-origin based | 2022 | 0 | < LOQ | / |
| **Tetramethrin** | Cereal-based | 2022 | 0 | < LOQ | / |
| **Tetramethrin** | Animal-origin based | 2023 | 0 | < LOQ | / |
| **Tetramethrin** | Cereal-based | 2023 | 4 | 0.02 | 0.02 |
| **Triadimenol** | Animal-origin based | 2019 | 0 | < LOQ | / |
| **Triadimenol** | Animal-origin based | 2020 | 0 | < LOQ | / |
| **Triadimenol** | Animal-origin based | 2021 | 0 | < LOQ | / |
| **Triadimenol** | Cereal-based | 2021 | 0 | < LOQ | / |
| **Triadimenol** | Animal-origin based | 2022 | 0 | < LOQ | / |
| **Triadimenol** | Cereal-based | 2022 | 0 | < LOQ | / |
| **Triadimenol** | Animal-origin based | 2023 | 0 | < LOQ | / |
| **Triadimenol** | Cereal-based | 2023 | 3 | 0.03 | 0.01 |
| **Trifluralin** | Animal-origin based | 2019 | 0 | < LOQ | / |
| **Trifluralin** | Animal-origin based | 2020 | 1 | 0.04 | / |
| **Trifluralin** | Animal-origin based | 2021 | 0 | < LOQ | / |
| **Trifluralin** | Cereal-based | 2021 | 0 | < LOQ | / |
| **Trifluralin** | Animal-origin based | 2022 | 0 | < LOQ | / |
| **Trifluralin** | Cereal-based | 2022 | 0 | < LOQ | / |
| **Trifluralin** | Animal-origin based | 2023 | 0 | < LOQ | / |
| **Trifluralin** | Cereal-based | 2023 | 0 | < LOQ | / |
